# Feasibility and Safety of Perilla Seed Oil as an Additional Antioxidative Therapy in Patients with Mild to Moderate Dementia

**DOI:** 10.1155/2018/5302105

**Published:** 2018-06-04

**Authors:** Chuntida Kamalashiran, Junya Pattaraarchachai, Sombat Muengtaweepongsa

**Affiliations:** ^1^Chulabhron International College of Medicine, Thammasat University, Pathum Thani, Thailand; ^2^Faculty of Medicine, Thammasat University, Pathum Thani, Thailand

## Abstract

Dementia is a broad-spectrum terminology for a degenerate in cognitive function severe enough to intervene in activities of daily living. Oxidative stress plays a major role in the neurodegenerative cascade, leading to the irreversible mechanism in dementia. Perilla seed oil is extracted from its seeds and contains a high source of antioxidative substances such as omega-3 fatty acid. With its prominent antioxidative property, perilla seed oil demonstrates neuroprotective effects against dementia in preclinical studies. We aim to prove the feasibility and safety of perilla seed oil as an additional antioxidative therapy in patients with dementia. This single-centered, double-blinded, placebo-controlled trial randomized 239 patients with clinical diagnosis of mild to moderate dementia according to the Thai Mini-Mental State Examination (TMSE) score of 10 to 23 or the Thai Montreal Cognitive Assessment score of 12 to 25. Either two capsules containing 500 milligrams of perilla seed oil or similarly appearing two capsules containing 500 milligrams of olive oil (placebo) four times daily was added to conventional standard treatment of dementia for six months. Clinical side effects and routine laboratory results at baseline and after treatment were compared between both groups. Nausea and vomiting were the most common clinical side effects (3%) found equally in both groups. Three patients in the placebo group prematurely discontinued the medication, while only one patient in the treatment group quit the medication early. However, about 5% of patients in both groups could not comply with the regimen of the treatment. The routine laboratory results, including complete blood counts, kidney function tests, and liver function panels, at baseline and after treatment, were not significantly different in both groups. In conclusion, perilla seed oil was feasible and safe to add on with standard treatment in patients with mild to moderate dementia. Further study is needed to confirm its benefit to use as additional antioxidative therapy in patients with dementia.

## 1. Introduction

Dementia is a broad-spectrum terminology for a degenerate in cognitive function severe enough to intervene in activities of daily living [1]. The most common subtypes of dementia include Alzheimer disease, vascular dementia, frontotemporal dementia, dementia with Lewy body, and dementia in Parkinson's disease [2]. Prevalence of dementia increases with age [3]. The age-specific prevalence of dementia is duplicated with every five-year increase in individuals aged 65 [4, 5]. In the United States, the prevalence of dementia was 5% in people aged between 71 and 79, but it raised to 37.4% in people older than 90 [6]. According to a cross-sectional national survey in 2001, the prevalence of dementia among the Thai population increased from 1% of elderly aged 60–64 to 31.3% of elderly aged 90 or older [7].

Dementia is a degenerative and irreversible brain disease [8]. Oxidative stress plays a major role in the neurodegenerative cascade, leading to the irreversible mechanism in dementia [9, 10]. Oxidative stress leads to beta-amyloid deposit in the brain tissue and cerebral vessels [11]. Perilla oil is extracted from its seeds and contains a high source of antioxidative substances such as omega-3 fatty acid [12]. With its prominent antioxidative property, perilla seed oil demonstrates neuroprotective effects against dementia in preclinical studies [13]. Learning ability and memory improved in rats fed with perilla seed oil diet [14]. We aim to prove the feasibility and safety of perilla seed oil as an additional antioxidative therapy in patients with dementia.

## 2. Materials and Methods

This is a single-centered, double-blinded, placebo-controlled trail. Patients with mild to moderate dementia who visited dementia clinic at Thammasat University Hospital from January 2014 to December 2016 were enrolled in the study. Routine clinical examination, laboratory results, and neuroimaging per protocol for diagnosis of dementia were performed. The Thai Mini-Mental State Examination (TMSE) score [15] and/or the Thai Montreal Cognitive Assessment (MoCA) score [16] were completed. Patients with treatable etiologies, such as hypothyroidism, vitamin B12 deficiency, and normal pressure hydrocephalus, were excluded. Mild to moderate dementia is defined by the Thai Mini-Mental State Examination (TMSE) score of less than 20 or the Thai Montreal Cognitive Assessment (MoCA) score of less than 23. Inclusion and exclusion criteria are shown in Table 1.

Dementia subtypes were diagnosed based on clinical features, laboratory results, and imaging findings [17, 18]. The patients were 1 : 1 randomized and divided into 2 groups. Perilla seed oil or placebo (olive oil), taking 2 capsules three times after meal, was added to conventional standard treatment of dementia for six months. Adverse effect and clinical features were reported. The study was registered under the Thai Clinical Trials Registry: register number 142/2556.

## 3. Results

**Figure 1:**
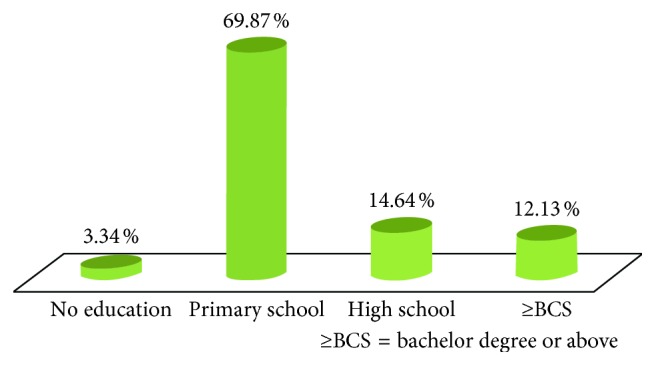
Education level.

A total of 239 patients with mild to moderate dementia were enrolled in the study. Proportion of either gender was almost equal. Mean age was 76 years old. Most of the patients had completed primary school. The education level of participants is shown in Figure 1. The most common preexisting disease was hypertension. Demographic data are shown in Table 2.

**Figure 2:**
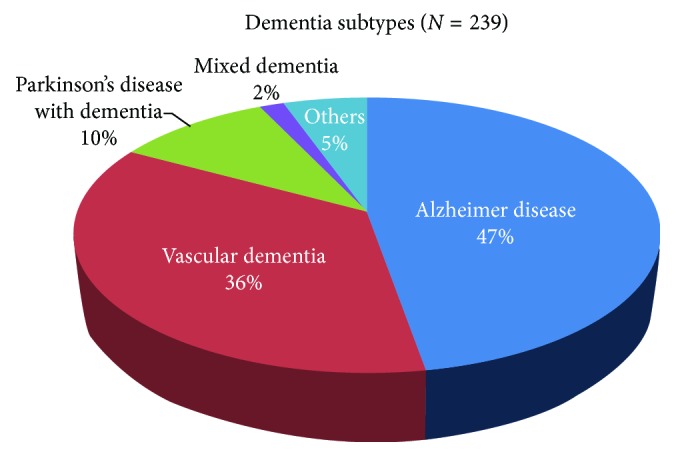
Dementia subtypes.

The most common subtype of dementia was Alzheimer disease, and the second most common subtype was vascular dementia. The subtypes of dementia are shown in Figure 2. Fifty-seven patients could not complete the study as they were not able to comply with the regimen of the treatment. The most common reasons for noncompliance were unable to follow the regimen three times daily. This was accounted for almost 25% of all participants. The remaining, 182 patients, were able to complete the study. The subtypes of dementia in 182 patients enrolled to the study and in 57 patients dropped out from the study are shown in Table 3. There was no statistical significance between Alzheimer and non-Alzheimer subgroups in both the enrolled and drop-out groups (Table 4).

Nausea and vomiting were the most common clinical side effects (3%) found equally in both groups. Three patients in the placebo group prematurely discontinued the medication, while only one patient in the treatment group quit the medication early. However, about 5% of patients in both groups could not comply with the regimen of the treatment. The routine laboratory results, including complete blood counts, kidney function tests, and liver function panels, at baseline and after treatment, were not significantly different in both groups.

## 4. Discussion

Perilla seed oil appears to be safe to add on with the conventional treatment in patients with mild to moderate dementia. Natural ingredients of perilla seed oil seem to be nontoxic [19]. However, as many as 57 patients or almost one-fourth of participants did not comply with the high frequency of medical ingestion with 2 capsules three times daily. This may imply that three times daily dosage is not appropriate to be applied in patients with dementia. Enteric coating formulation should help improve bioavailability and reduce frequency of administration of perilla seed oil treatment [20]. Perilla seed oil in a soft gelatin capsule can be taken in twice daily regimen and is now available in the market [12].

Interaction between perilla seed oil and conventional medications for patients with dementia is unlikely [21]. Perilla seed oil contains remarkable amount of omega-3 fatty acid [12]. Interaction between omega-3 fatty acid and warfarin is still controversial [22, 23]. Patients who currently take vitamin K antagonist were initially excluded from the study due to potential interaction between perilla seed oil and warfarin. However, the interaction between perilla seed oil and warfarin needs to be confirmed by both in vitro and in vivo studies.

The most common subtypes of dementia in our study were Alzheimer disease and vascular dementia. This prevalence of dementia subtype is correlated with a previous clinicopathological study from a developing country [24]. The proportion of vascular dementia in developing countries including in our study is higher than that in developed countries, while there is no difference in the proportion of Alzheimer disease in either population [24, 25]. This finding may represent the poor control of vascular risk factors in developing countries.

Relationship between education level and dementia is controversial. Many studies demonstrated that lesser educational accomplishment was related to higher occurrence of dementia [26]. Patients with low education level begin with lesser cognitive reserve and then end up with lower ability to deal with dementia [27]. In contrast, higher education may lead to faster cognitive decline in patients with Alzheimer disease and vascular dementia [28, 29].

The neuroprotective therapy is a kind of disease modification treatment which is helpful for outcomes improvement [30]. The preclinical study demonstrated neuroprotective effects of perilla seed oil in the guinea pig [31]. The neuroprotective therapy is lacking in the present formula of conventional treatment for patients with mild to moderate dementia [32]. Therefore, add-on treatment with neuroprotective therapy should provide some benefits. However, a clinical study is needed to demonstrate the exact benefits in human.

## 5. Conclusions

Perilla seed oil was feasible and safe to add on with standard treatment in patients with mild to moderate dementia. Further study is needed to confirm its benefit to use as additional antioxidative therapy in patients with mild to moderate dementia.

## Figures and Tables

**Table 1:** Inclusion and exclusion criteria.

*Inclusion criteria*
1. Age > 50–90 years
2. Screening cognitive function by the Thai Mental State Examination (TMSE) between 10 and 23 and/or MoCA between 7 and 20
3. Diagnosing mild to moderate dementia by following the Diagnosis and Statistical Manual of Mental Disorder, Fourth Edition (DSM-IV)
4. Can recognize and communicate
5. Do not have severe complications that influence patient cooperation with this study
6. Willing to participate in this study and conducts oneself follow by suggestion duration in this study
7. Volunteer or proxy is able to provide the consent

*Exclusion criteria*
1. Serum creatinine higher than 2 mg/dL
2. Serum alanine aminotransferase (ALT), aspartate aminotransferase (AST), total bilirubin, or alkaline phosphatase higher than 2.5 times of the upper normal range
3. Vitamin B12 deficiency
4. Hypothyroidism
5. Syphilis
6. Bleeding disorder or taking warfarin
7. Major depressive disorder
8. History of medicinal and herbal hypersensitivities
9. Having severe illness history
10. Intake of other supplements

**Table 2:** Demographic data (*n*=239).

Age, mean (years-old)	76.2
Gender	
Male (%)	118 (49)
Female (%)	121 (51)
Educational level	
No education (%)	8 (3.34)
Primary school (%)	167 (69.87)
Secondary school (%)	35 (14.64)
Bachelor degree or above (%)	29 (12.13)
Comorbid conditions	
Hypertension (%)	129 (53.97)
Diabetes (%)	51 (21.33)
Hyperlipidemia (%)	32 (13.38)
Heart disease (%)	2 (0.83)
Smoking (%)	37 (15.48)
Alcohol drinking (%)	38 (15.89)
History of head injury (%)	15 (6.27)
Family history of dementia (%)	37 (15.48)

**Table 3:** Patients enrolled to the study and dropped out from the study divided by subtypes of dementia.

Volunteers according to the type of dementia enrolled	*N*=182	Alzheimer's disease (*N*=86)	Vascular dementia (*N*=68)	Parkinson's disease with dementia (*N*=18)	Mixed dementia (*N*=3)	Other dementia (*N*=7)
Perilla seed oil	94 (51.65)	49 (56.98)	32 (47.06)	8 (44.44)	2 (66.66)	3 (42.86)
Placebo	88 (48.35)	37 (43.02)	36 (52.94)	10 (55.55)	1 (33.33)	4 (57.14)
*Volunteers according to the type of dementia drop-out*	*N*=57	*Alzheimer's disease*	*Vascular dementia*	*Parkinson's disease with dementia*	*Mixed dementia*	*Other dementia*
Perilla seed oil	27 (47.37)	15 (55.55)	6 (22.22)	3 (11.11)	1 (3.70)	2 (7.41)
Placebo	30 (52.60)	12 (40.00)	11 (36.66)	3 (10.00)	0 (0.00)	4 (13.33)

**Table 4:** Comparison between Alzheimer and non-Alzheimer subtypes in enrolled and drop-out groups.

Volunteers according to the type of dementia enrolled (*N*=182)	Alzheimer's disease	Non-Alzheimer's disease	*P* value
Perilla seed oil	49 (52.17%)	45 (47.87%)	0.173
Placebo	37 (42.04%)	5 (58%)	
*Volunteers according to the type of dementia drop-out (N*=57)			
Perilla seed oil	15 (55.5%)	12 (44.4%)	0.24
Placebo	12 (40%)	18 (60%)	

## Data Availability

The data used to support the findings of this study are available from the corresponding author upon request.
